# Compromised fertility disrupts *Peg1* but not *Snrpn* and *Peg3* imprinted methylation acquisition in mouse oocytes

**DOI:** 10.3389/fgene.2012.00129

**Published:** 2012-07-11

**Authors:** Michelle M. Denomme, Carlee R. White, Carolina Gillio-Meina, William A. MacDonald, Bonnie J. Deroo, Gerald M. Kidder, Mellissa R. W. Mann

**Affiliations:** ^1^Children’s Health Research Institute,London, ON, Canada; ^2^Department of Obstetrics and Gynaecology, Schulich School of Medicine and Dentistry, University of Western Ontario,London, ON, Canada; ^3^Department of Biochemistry, Schulich School of Medicine and Dentistry, University of Western Ontario,London, ON, Canada; ^4^Department of Physiology and Pharmacology, Schulich School of Medicine and Dentistry, University of Western Ontario,London, ON, Canada; ^5^Department of Oncology, Schulich School of Medicine and Dentistry, University of Western Ontario,London, ON, Canada

**Keywords:** genomic imprinting, DNA methylation, imprint acquisition, infertility, oocyte, connexin37, estrogen receptor beta, oocyte diameter

## Abstract

Growth and maturation of healthy oocytes within follicles requires bidirectional signaling and intercellular gap junctional communication. Aberrant endocrine signaling and loss of gap junctional communication between the oocyte and granulosa cells leads to compromised folliculogenesis, oocyte maturation, and oocyte competency, consequently impairing fertility. Given that oocyte-specific DNA methylation establishment at imprinted genes occurs during this growth phase, we determined whether compromised endocrine signaling and gap junctional communication would disrupt *de novo* methylation acquisition using ERβ and connexin37 genetic models. To compare mutant oocytes to control oocytes, DNA methylation acquisition was first examined in individual, 20–80 μm control oocytes at three imprinted genes, *Snrpn, Peg3*, and *Peg1*. We observed that each gene has its own size-dependent acquisition kinetics, similar to previous studies. To determine whether compromised endocrine signaling and gap junctional communication disrupted *de novo* methylation acquisition,**individual oocytes from *Esr2-* and *Gja4*-deficient mice were also assessed for DNA methylation establishment. We observed no aberrant or delayed acquisition of DNA methylation at *Snrpn, Peg3*, or *Peg1 *in oocytes from *Esr2-*deficient females, and no perturbation in *Snrpn *or* Peg3* *de novo* methylation in oocytes from *Gja4*-null females. However, *Gja4* deficiency resulted in a loss or delay in methylation acquisition at *Peg1*. One explanation for this difference between the three loci analyzed is the late establishment of DNA methylation at the *Peg1 *gene. These results indicate that compromised fertility though impaired intercellular communication can lead to imprinting acquisition errors. Further studies are required to determine the effects of subfertility/infertility originating from impaired signaling and intercellular communication during oogenesis on imprint maintenance during preimplantation development.

## INTRODUCTION

The tight regulation of monoallelic gene expression based on gametic origin is termed genomic imprinting (Bartolomei and Ferguson-Smith, 2011). This dynamic process relies on epigenetic modifications such as DNA methylation to mark, or “imprint”, one of the two parental alleles, resulting in differential gene expression in progeny (Verona et al., 2003). Gametogenesis encompasses the critical period of heritable epigenetic reprogramming for imprinted genes. Imprinted DNA methylation is first erased in primordial germ cells, subsequently allowing for *de novo* differential methylation at imprinted loci in oocytes and sperm (Li and Sasaki, 2011). In males, *de novo* DNA methylation acquisition occurs during the prenatal stages of spermatogenesis, beginning in prospermatogonia and is completed by birth (Kafri et al., 1992; Davis et al., 1999, 2000; Ueda et al., 2000). In females, *de novo* DNA methylation is acquired after oocytes enter the growth phase of follicular development, from the primary to antral follicle stage (Lucifero et al., 2004; Hiura et al., 2006; Sato et al., 2007; Song et al., 2009). Importantly for oocytes, imprinted methylation acquisition is dependent on oocyte size and not oocyte age, with methylation levels increasing as oocyte diameter increases.

The correct establishment of germline imprints is significant as disruptions to this process can result in the development of imprinting disorders such as Beckwith–Wiedemann syndrome (BWS), Silver–Russell Syndrome (SRS), and Angelman syndrome (AS). BWS is an overgrowth disorder that is caused by imprinting defects that result in a gain of maternal methylation at the *H19* imprinting control region (ICR) or a loss of maternal-specific methylation at the *KCNQ1OT1* (*KCNQ1 overlapping transcript 1*) ICR (Weksberg et al., 2010). SRS is an intrauterine growth restricted imprinting disorder with imprinting defects at the *H19* and possibly at the *paternally expressed gene 1* (*Peg1*) imprinted domains (Eggermann, 2010). AS is a neurological disorder that is caused by loss of maternal-specific methylation at the *small nuclear ribonucleoprotein N* (*SNRPN*) ICR (Mabb et al., 2011). Sporadic epigenetic errors resulting in these disorders are reported to occur more frequently in the assisted reproductive technologies (ARTs) population (Cox et al., 2002; DeBaun et al., 2003; Gicquel et al., 2003; Maher et al., 2003; Orstavik et al., 2003; Halliday et al., 2004; Chang et al., 2005; Ludwig et al., 2005; Rossignol et al., 2006; Azzi et al., 2009; Bliek et al., 2009; Lim et al., 2009; Lennerz et al., 2010; Turner et al., 2010). For AS, patients at the highest risk for an imprinting defect have parents with prolonged infertility undergoing infertility treatment (Ludwig et al., 2005; Doornbos et al., 2007). This raises the question as to whether imprinting errors in ART patients are associated with parental infertility/subfertility. While studies have been conducted to determine the effects of ARTs on genomic imprinting, investigations of how impaired fertility may contribute to imprinting errors are lacking. In this study, we queried whether impaired fertility arising during oogenesis could lead to imprinting defects.

Development of healthy oocytes is dependent on interactions between the growing oocyte and surrounding follicular cells (Kidder and Vanderhyden, 2010). Oocytes play an important role in regulating granulosa cell development, proliferation, and differentiation, as well as steroid hormone production. In turn, follicular cells play a critical role in oocyte growth, meiotic progression, and transcriptional activity and chromatin remodeling of the oocyte genome. This synergistic partnership is facilitated by endocrine and paracrine signaling, and intercellular gap junctional communication, ensuring meiotic and developmental competence of the oocyte. In this study, we specifically examined the effects of aberrant signaling and communication on imprint acquisition.

A complex endocrine signaling pathway is active in the ovary that regulates follicle and oocyte development. 17β-estradiol acting through nuclear estrogen receptor beta (ERβ) augments the actions of follicle stimulating hormone (FSH). In the ovary, ERβ is expressed primarily in granulosa cells and at low levels in the oocyte (Drummond and Fuller, 2011). Female mice bearing a targeted deletion of the ERβ (*Esr2*) gene are subfertile, producing fewer oocytes following superovulation, as well as litters with fewer pups (Krege et al., 1998; Couse et al., 2000, 2003, 2005; Dupont et al., 2000; Emmen et al., 2005). Attenuated differentiation of granulosa cells following gonadotropin stimulation in *Esr2-*null mice leads to decreased antrum formation, delayed follicle maturation, and reduced follicular rupture, producing greater numbers of atretic follicles and fewer preovulatory oocytes. In addition, vascularization of the thecal layer, which is required for follicular growth, is impaired (Inzunza et al., 2007). Mechanistically, ERβ is required for optimal cAMP production in mouse granulosa cells following gonadotropin stimulation (Deroo et al., 2009). ERβ deficiency causes disruption of cAMP second messenger signaling in granulosa cells in response to FSH, producing aberrant FSH-regulated gene expression, decreased response to luteinizing hormone, and impaired ovulation and fertility.

Gap junctions are specialized channels composed of six membrane proteins termed connexins (CX). These channels are essential for communication between neighboring cells (Harris, 2001). In the mouse, CX37 and CX43 are the only connexins known to be required in developing follicles (Kidder and Vanderhyden, 2010). CX43 localizes to gap junctions in the granulosa cell membranes, enabling granulosa cell to granulosa cell communication. By comparison, CX37 constitutes the gap junctions coupling the oocyte with surrounding granulosa cells and is specifically located at the interface between the oocyte and the first layer of granulosa cells (Simon et al., 1997). Gap junctions allow the transport of nutrients, metabolites and second messengers, such as cAMP, between the granulosa cells and the oocyte (Kidder and Vanderhyden, 2010). Targeted deletion of the CX37 (*Gja4*) gene causes arrested folliculogenesis at the early antral stage, impaired oocyte development and meiotic competency, and premature luteinization of the follicles (Simon et al., 1997; Carabatsos et al., 2000).

In this study, we employed the *Esr2*^–/–^ and *Gja4*^–/–^ genetic models to interfere specifically with endocrine signaling and gap junctional communication, compromising fertility. We hypothesized that inhibition of the ERβ pathway and/or oocyte–granulosa cell gap junctional communication would lead to perturbations in imprinted methylation acquisition. To compare mutant oocytes to control oocytes, DNA methylation acquisition was first examined in individual, 20–80 μm diameter control oocytes at three imprinted genes, *Snrpn, Peg3*, and *Peg1* (also known as *Mest*). Similar to previous studies (Lucifero et al., 2004; Hiura et al., 2006; Sato et al., 2007; Song et al., 2009), we observed that each gene had its own size-dependent acquisition kinetics. To determine whether compromised endocrine signaling and gap junctional communication disrupted *de novo* methylation acquisition,** preovulatory oocytes from *Esr2*^–/–^ females, and early antral stage oocytes from *Gja4*^–/–^ mice were assessed for DNA methylation establishment at *Snrpn, Peg3*, and *Peg1*. We observed no aberrant or delayed acquisition of DNA methylation at *Snrpn, Peg3*, and *Peg1* in preovulatory oocytes from ERβ-deficient females. Similarly, we found no perturbation of *Snrpn* and* Peg3* *de novo* methylation in oocytes from CX37-null follicles. However, *Peg1* methylation acquisition was lost or delayed in *Gja4*-deficient oocytes compared to controls. We attribute this to the late establishment of DNA methylation at the *Peg1* gene. These results indicate that compromised fertility though impaired intercellular communication can lead to imprinting acquisition errors. Further studies are required to determine the post-fertilization effects of subfertility/infertility originating from impaired signaling and intercellular communication during oogenesis.

## MATERIALS AND METHODS

### OOCYTE ISOLATION

#### Control oocyte collections

Ovaries were obtained from C57BL/6 female mice (Charles River) at 10, 14, 21, and 28 days postpartum (dpp), and placed in Waymouth MB 752/1 medium (Invitrogen) supplemented with 10% fetal bovine serum (Li et al., 2007). For further follicle separation, ovaries were digested in the same medium containing 2 mg/ml type I collagenase (Sigma-Aldrich) at 37°C. Primary, secondary and early tertiary (antral) follicles were liberated by repeated aspiration and expulsion with a 1 ml pipette. Follicles were washed several times in culture medium without collagenase. For oocyte isolation, follicles were centrifuged for 5 min at 4,000 rpm, re-suspended and digested in 0.05% Trypsin/EDTA in a culture dish for 15 min at 37°C. Oocytes were dissociated from the granulosa cells by repeated aspiration and expulsion with a 1 ml pipette. Oocytes were retrieved through mouth pipetting and placed in 30 μl drops of M2 medium (Sigma) for further analysis.

#### Gja4-null oocyte collections

Ovaries were removed from *Gja4*^–/–^ female mice (C57BL/6 background) at 21 and 28 dpp, and placed in Waymouth MB 752/1 medium (Invitrogen) supplemented with 10% fetal bovine serum. *Gja4-*null oocytes were retrieved through the same collection method as control oocytes and placed in 30 μl M2 medium (Sigma) for further analysis.

#### Esr2-null oocyte collections

Ovaries were removed from *Esr2*^–/–^ females (C57BL/6 background) at 28 dpp and placed in a 100-mm cell culture dish containing 15 ml ice-cold M199 medium (Sigma) supplemented with 1 mg/ml BSA (Invitrogen) and 2.5 g/ml gentamicin (Invitrogen; Deroo et al., 2009). Follicles were released by manual puncture with 25-gauge needles and subsequent pressure applied with a sterile spatula. Oocytes were retrieved through mouth pipetting and transferred to 30 μl drops of M2 medium (Sigma) for further analysis.

#### Single oocyte bisulfite mutagenesis and sequencing

Processing, embedding, and bisulfite mutagenesis of individual oocytes was performed as previously described (Denomme et al., 2011). Briefly, oocytes were treated with 0.3 mg/ml hyaluronidase (Sigma) to remove any surrounding cumulus cells (if present), washed three times in 30 μl drops of M2 medium (Sigma), and then imaged using the Olympus IX81 microscope. Oocyte diameter was measured using Macnification v.1.8 (Orbicule). Following treatment with acidic Tyrode’s solution (Sigma) to remove the zona pellucida (if present), oocytes were washed twice in M2 medium, then individual oocytes were embedded in 10 μl of 2:1 LMP agarose and lysis solution [100 mM Tris–HCl, pH 7.5 (Bioshop), 500 mM LiCl (Sigma), 10 mM EDTA, pH 8.0 (Sigma), 1% LiDS (Bioshop), and 5 mM DTT (Sigma), 1 μl 2 mg/ml proteinase K (Sigma), and 1 μl 10% Igepal (Sigma)] under 300 μl of mineral oil (Sigma), and placed on ice for 10 min for the agarose to harden. Mineral oil was replaced with 500 μl SDS lysis buffer [450 μl 1× Tris EDTA (TE), pH 7.5 [10 mM Tris (Bioshop), 1 mM EDTA], 50 μl 10% SDS (Bioshop), 1 μl 2 mg/ml proteinase K] and incubated at 50°C overnight. Following overnight incubation, lysis buffer was replaced with 300 μl mineral oil and oocytes were either immediately treated for bisulfite conversion or frozen at -20°C for up to 5 days. Firstly, samples were placed at 90°C for 2.5 min to heat inactivate the proteinase K, and then DNA was denatured using 0.1 M NaOH (Sigma) at 37°C for 15 min. Treatment with 2.5 M bisulfite solution (0.125 M hydroquinone (Sigma), 3.8 g sodium hydrogen sulfite (Sigma), 5.5 ml water, and 1 ml 3 M NaOH) at 50°C for 3.5 h was followed by desulfonation using 0.3 M NaOH at 37°C for 15 min. Samples were washed twice in 1× TE pH 7.5 and twice in water, and then added directly to a Ready-To-Go PCR bead (GE) consisting of 15 μl water, gene-specific primers and 1 μl of 240 ng/ml transfer RNA as a carrier, with 25 μl mineral oil overlay. Negative controls (no oocyte) were processed alongside each bisulfite reaction. PCR amplification of the *Snrpn* ICR, *Peg3* DMR, and *Peg1* DMR was performed as previously described (Market-Velker et al., 2010). Following ligation into the PGEM-T easy vector (Promega) and cloning, 30 μl of colony PCR product was sent to Bio-Basic Inc. (Markham, Ontario, Canada) for sequencing. For each sample, five clones were sequenced. As MI oocytes have not extruded the first polar body, both alleles were successfully amplified in some oocytes, and only one allele was detectable in other oocytes. However, oocytes with more than two clones having very different methylation patterns and different non-CpG conversion rates were excluded from analysis, as cumulus cell contamination could not be ruled out. **Table 1** gives the number of oocytes included and excluded from analysis per gene.

**Table 1:** TABLE 1. Number of oocytes included and excluded from analysis

	*Snrpn*			*Peg3*			*Peg1*		
	IN	EX	%EX	IN	EX	%EX	IN	EX	%EX
WT	55	1	1.8	56	6	9.7	58	7	11.1
*Esr2*^–/–^	12	1	7.7	11	1	8.3	11	1	8.3
*Gja4*^–/–^	31	5	13.9	20	1	4.8	31	3	8.8
Total	98	7	6.7	87	8	8.4	100	11	9.9

*IN, included oocytes with one to two clone patterns; EX, excluded oocytes with 3 or more clone patterns.*

### STATISTICAL ANALYSIS

For each imprinted gene, significant difference of CpG methylation percentage was determined by a two-tailed Mann–Whitney *U* test between mutant oocytes and control oocytes matched for size. A diameter range of 65–80 μm was used to compare *Esr2*-deficient oocytes to control oocytes, while the 35–60 μm diameter range (including KO468 for *Snrpn* with a diameter of 60.5 μm) was used to compare the *Gja4* deficient to control oocytes. A *p*-value of <0.05 was taken to be statistically significant.

## RESULTS

### METHYLATION ACQUISITION IN CONTROL OOCYTES CORRELATES WITH OOCYTE DIAMETER

In female mammals, imprinted DNA methylation has been shown to arise during follicle growth from the primary to the antral stage in correlation with oocyte diameter (Lucifero et al., 2004; Hiura et al., 2006), with gene-specific kinetics for imprint acquisition. However, these analyses were performed with pooled oocytes of different sizes. To compare individual mutant oocytes to control oocytes, we first needed to examine imprinted DNA methylation acquisition in individual control oocytes. C57BL/6 oocytes were collected at 10, 14, 21, and 28 dpp to obtain oocytes with a diameter range of 20–80 μm. Oocytes that were collected at 10 dpp displayed a diameter range of 20–70 μm, those at 14 dpp were 40–80 μm in diameter, at 21 dpp ranged from 50 to 70 μm, and at 28 dpp ranged from 60 to 80 μm in diameter.

**FIGURE 1:**
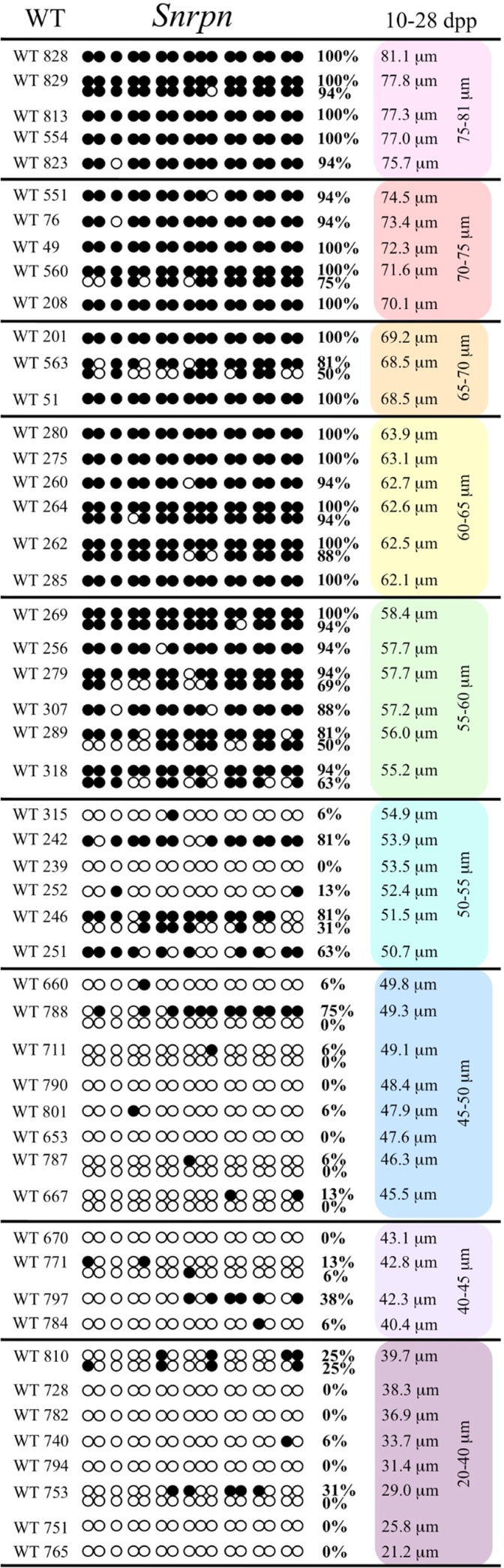
**Methylation analysis of the *Snrpn* ICR in individual oocytes derived from control C57BL/6 female mice.** The *Snrpn* ICR region analyzed contains 16 CpGs. Black circles indicate methylated CpGs while white circles indicate unmethylated CpGs. Each row represents an individual oocyte (designation indicated to the left). Methylation percentage and diameter for each oocyte is shown at the right. Oocytes are grouped into cohorts ranging from 20 to 80 μm diameters in 5 μm increments. Oocytes with one methylation pattern represent one of the two parental alleles detected. Oocytes with two methylation patterns represent detection of both parental alleles.

**FIGURE 2:**
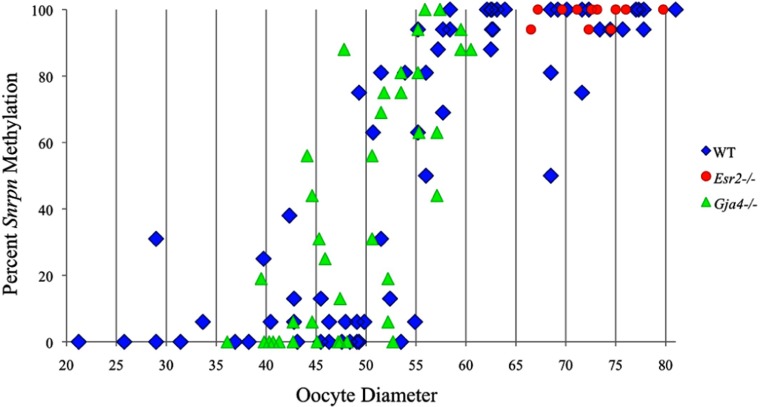
**Methylation percentage of each parental allele at the *Snrpn* ICR in relation to oocyte diameter (μm).** For oocytes with two parental alleles, each allele was graphed separately. Blue diamonds represent oocytes from control females, red circles represent oocytes from *Esr2*^–/–^ females, and green triangles represent oocytes from *Gja4*^–/–^ females.

**FIGURE 3:**
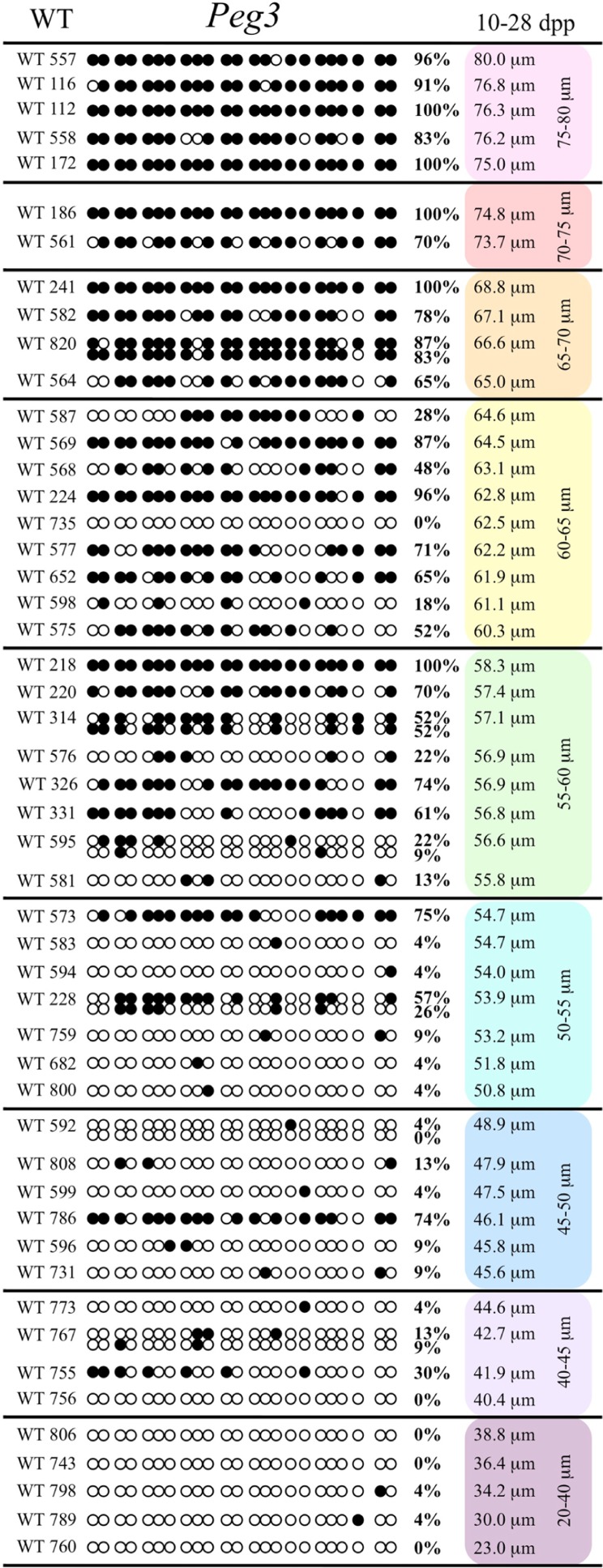
**Methylation analysis of the *Peg3* DMR in individual oocytes derived from control C57BL/6 females.** The *Peg3* DMR region analyzed contains 23 CpGs. Details are described in **Figure 1**.

**FIGURE 4:**
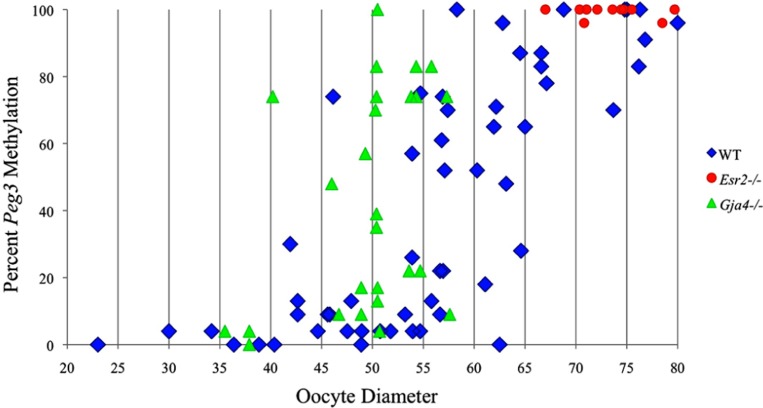
**Methylation percentage of each parental allele at the *Peg3* DMR in relation to oocyte diameter (μm).** Oocytes from control females, *Esr2*^–/–^ females and *Gja4*^–/–^ females are represented by blue diamonds, red circles and green triangles, respectively.

**FIGURE 5:**
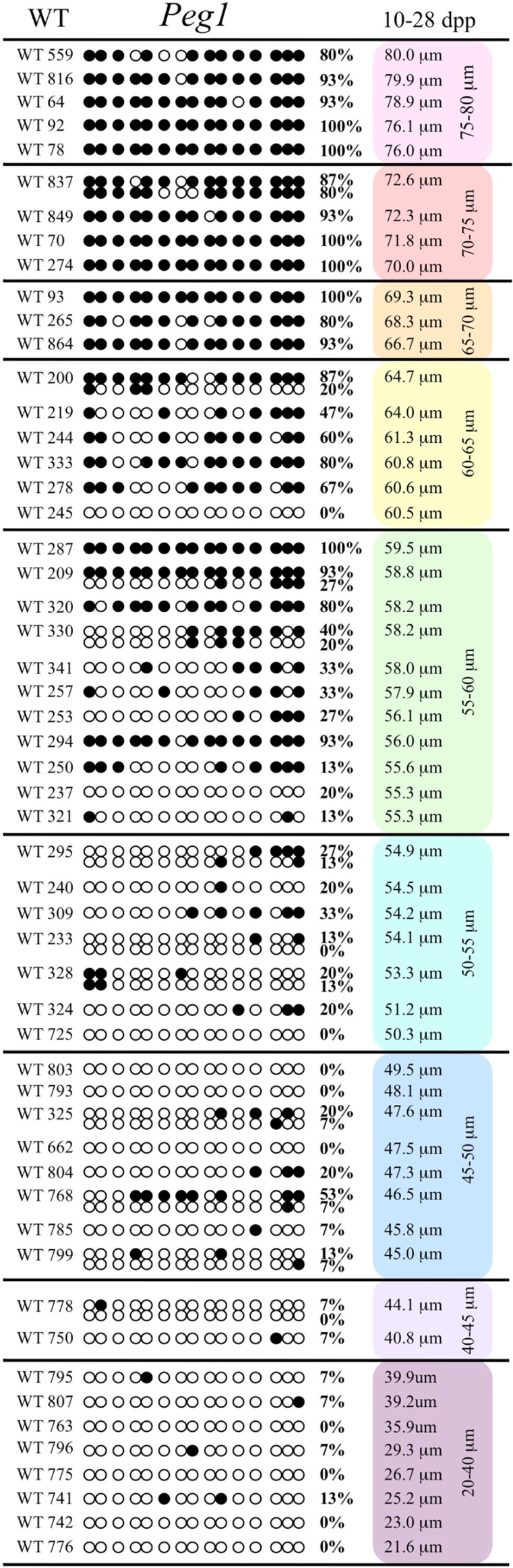
**Methylation analysis of the *Peg1* DMR in individual oocytes derived from the control C57BL/6 mice.** The *Peg1* DMR region analyzed contains 15 CpGs. Details are described in **Figure 1**.

**FIGURE 6:**
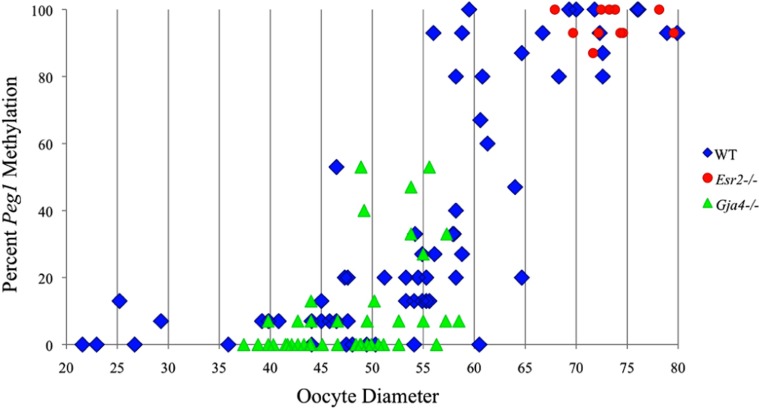
**Methylation percentage of each parental allele at the *Peg1* DMR in relation to oocyte diameter (μm).** Oocytes from control females, *Esr2*^–/–^ females and *Gja4*^–/–^ females are represented by blue diamonds, red circles and green triangles, respectively.

Analysis of *de novo* methylation acquisition at the *Snrpn* ICR showed mean methylation levels of 8.7% in 20–40 μm, 12.6% in 40–45 μm, 9.3% in 45–50 μm, 39.3% in 50–55 μm, 82.7% in 55–60 μm, 97.0% in 60–65 μm, 82.8% in 65–70 μm, 93.8% in 70–75 μm, and 98.0% in 75–80 μm oocytes (**Figures 1 and 2**). Likewise, mean methylation levels at the *Peg3* DMR were 1.6% in 20–40 μm, 11.2% in 40–45 μm, 16.1% in 45–50 μm, 22.9% in 50–55 μm, 47.5% in 55–60 μm, 51.7% in 60–65 μm, 82.6% in 65–70 μm, 85% in 70–75 μm, and 94.0% in 75–80 μm oocytes (**Figures 3 and 4**). For the *Peg1* DMR, mean methylation levels were 4.3% in 20–40 μm, 4.7% in 40–45 μm, 12.2% in 45–50 μm, 15.9% in 50–55 μm, 45.5% in 55–60 μm, 51.6% in 60–65 μm, 91.0% in 65–70 μm, 92.0% in 70–75 μm, and 93.2% in 75–80 μm oocytes (**Figures 5 and 6**). Thus, we observed that each gene had its own acquisition kinetics. DNA methylation acquisition began first for *Snrpn* at ~50 μm and was near completion at >60 μm. Next was *Peg3*, where DNA methylation acquisition was initiated at ~45 μm and nearly complete at >65 μm, which was followed by *Peg1*, where DNA methylation acquisition began at ~55 μm and was near completion by >70 μm. *Snrpn* had the shortest acquisition interval while *Peg3* had the longest.

### METHYLATION ACQUISITION IN ERβ-DEFICIENT OOCYTES

**FIGURE 7:**
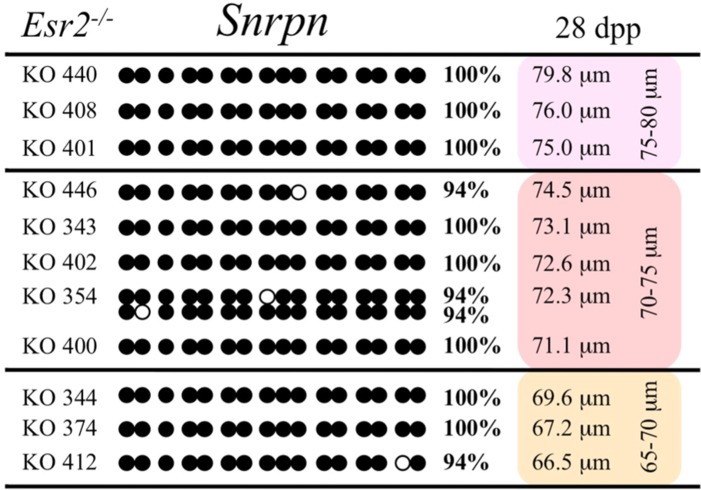
**Methylation analysis of the *Snrpn* ICR in individual oocytes derived from *Esr2*^–/–^ females.** Details are described in **Figure 1**.

**FIGURE 8:**
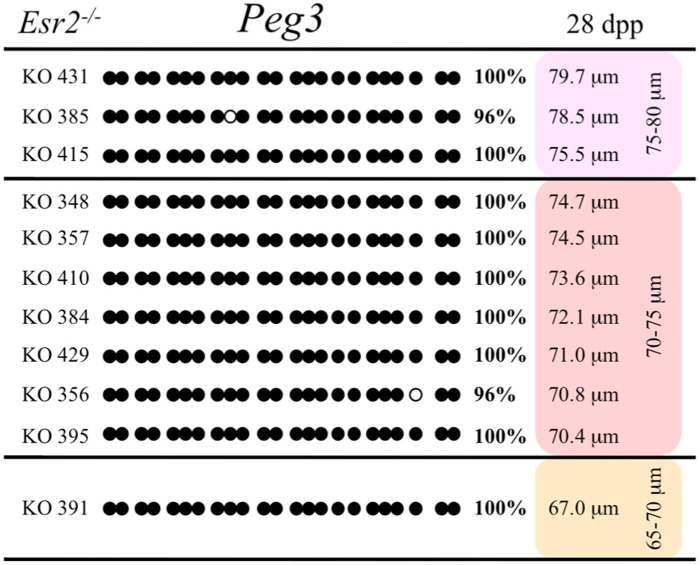
**Methylation analysis of the *Peg3* DMR in individual oocytes derived from *Esr2*^–/–^ mice.** Details are described in **Figure 1**.

**FIGURE 9:**
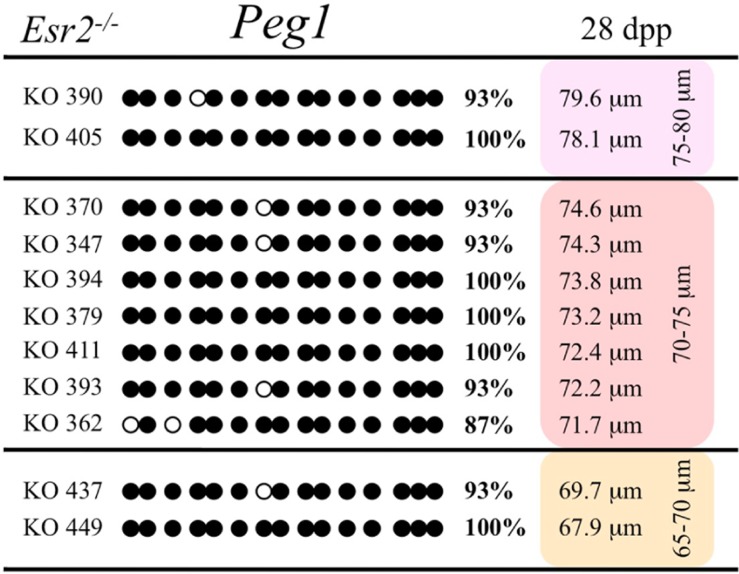
**Methylation analysis of the *Peg1* DMR individual oocytes derived from *Esr2*^–/–^ female mice.** Details are described in **Figure 1**.

Ovaries deficient in *Esr2* produce a reduced number of maturing oocytes, but those that do mature appear to not be developmentally compromised (Krege et al., 1998). Consistent with this, we recovered a small number of oocytes from 28 dpp females, ranging in diameter size from 66 to 82 μm, corresponding to the preovulatory stage in oocyte growth. To investigate the role of reduced hormone signaling on imprint acquisition, we analyzed the progression of DNA methylation acquisition in developing oocytes from mice deficient in *Esr2*. For the *Snrpn* ICR, mean methylation levels were 98.0% for 65–70 μm, 97.0% for 70–75 μm, and 100.0% for 75–80 μm oocytes (**Figures 2 and 7**). For the *Peg3* DMR, mean methylation was 100.0% in 65–70 μm, 99.4% in 70–75 μm, and 98.7% in 75–80 μm oocytes (**Figures 4 and 8**). For the *Peg1* DMR, mean methylation levels were 96.5% for 65–70 μm, 95.1% for 70–75 μm, and 96.5% for 75–80 μm oocytes (**Figures 6 and 9**). Thus, oocytes from *Esr2-*null females had comparable DNA methylation levels to control oocytes, indicating that imprint DNA methylation acquisition was unaffected by *Esr2* deficiency.

### METHYLATION ACQUISITION IN CX37-DEFICIENT OOCYTES

Previous analyses have shown that oocytes in CX37-null ovaries arrest development before reaching meiotic competence, around the time the antrum begins to form (~21 dpp; Simon et al., 1997; Carabatsos et al., 2000; Li et al., 2007). We collected and analyzed oocytes from *Gja4-*null** 21 dpp females, which ranged in diameter sizes from 35 to 55 μm and from 28 dpp females, which ranged in size from 50 to 60.5 μm. The maximum diameter obtained was 60.5 μm, consistent with previous studies (Simon et al., 1997; Carabatsos et al., 2000). To explore the relationship between gap junction loss and imprint acquisition, we analyzed the progression of DNA methylation establishment in developing oocytes from *Gja4*-deficient mice.

**FIGURE 10:**
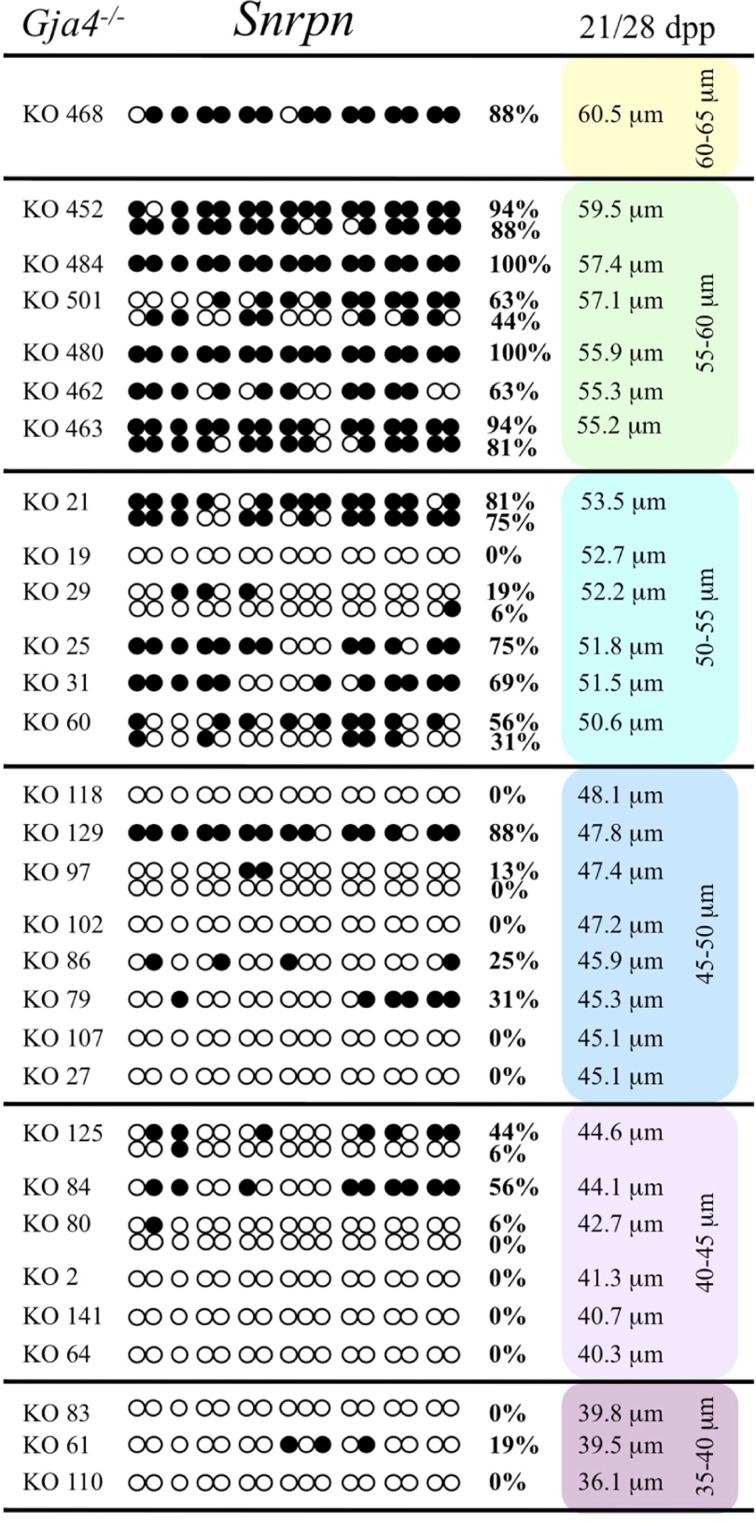
**Methylation analysis of the *Snrpn* ICR in individual oocytes derived from *Gja4*^–/–^ mice.** Details are described in **Figure 1**.

**FIGURE 11:**
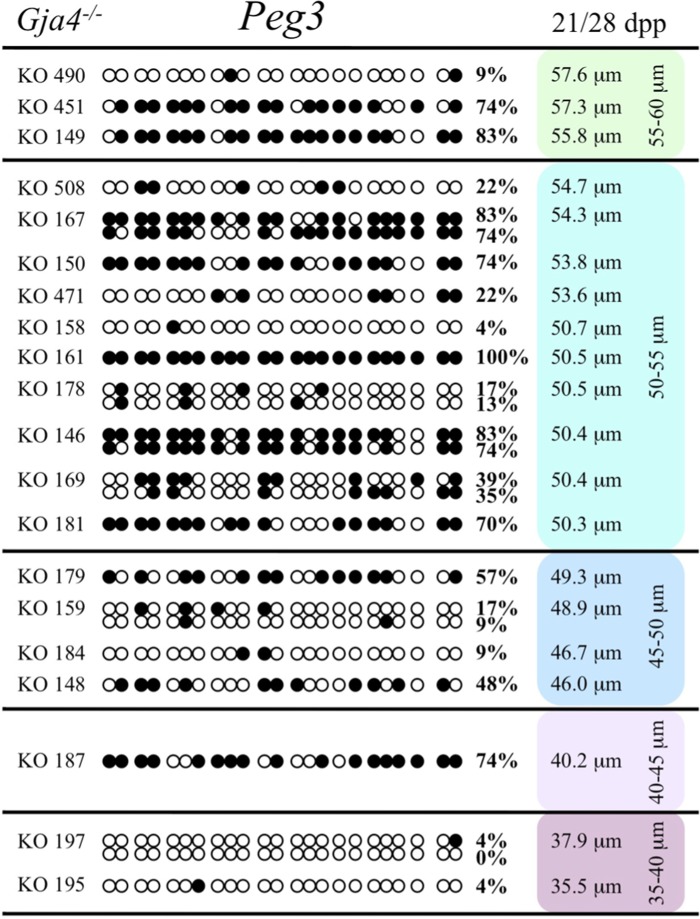
**Methylation analysis of the *Peg3* DMR individual GV oocytes derived from *Gja4*^–/–^ female mice.** Details are described in **Figure 1**.

**FIGURE 12:**
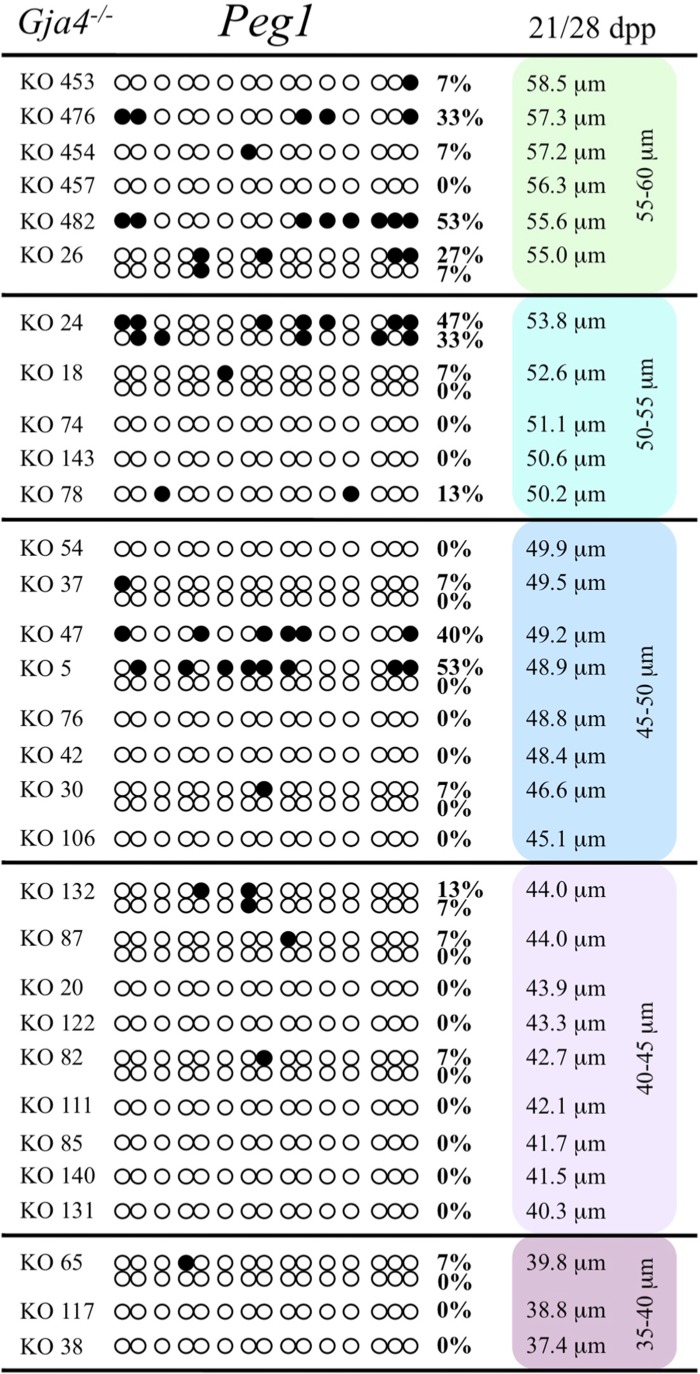
**Methylation analysis of the *Peg1* DMR individual GV oocytes derived from *Gja4*^–/–^ females.** Details are described in **Figure 1**.

At the *Snrpn* ICR, mean methylation levels were 6.3% in 35–40 μm, 14.0% in 40–45 μm, 17.4% in 45–50 μm, 45.8% in 50–55 μm, 80.8% in 55–60 μm, and 88.0% in 60–65 μm oocytes (**Figures 2 and 10**). No significant difference was observed in methylation levels between *Gja4-*null and control oocytes. Analysis at the *Peg3* DMR showed mean methylation levels of 2.7% for 35–40 μm, 74.0% in 40–45 μm, 28.0% for 45–50 μm, 50.7% for 50–55 μm, and 55.3% for 55–60 μm oocytes (**Figures 4 and 11**). No significant difference was observed in methylation levels between *Gja4*-null and control oocytes. For the *Peg1* DMR, mean methylation levels were 1.8% in 35–40 μm, 2.8% in 40–45 μm, 9.7% in 45–50 μm, 14.3% in 50–55 μm, and 19.1% in 55–60 μm oocytes (**Figures 6 and 12**). Statistical analysis of *Peg1* showed a significant difference in methylation acquisition between control and *Gja4*-deficient oocytes (*P* = 0.0006). Because *Gja4*-null oocytes stop growing and are eventually lost from the follicles, it could not be determined whether this is a delay or a disruption in *Peg1* DNA methylation acquisition.

## DISCUSSION

Growth and maturation of oocytes within follicles requires bidirectional signaling and exchange of nutrients, metabolites, and second messengers through gap junctions between the oocyte and granulosa cells (Matzuk et al., 2002; Gilchrist et al., 2008; Su et al., 2009). Aberrant endocrine signaling and loss of gap junctional communication between the oocyte and granulosa cells leads to compromised folliculogenesis, oocyte maturation, and oocyte competency, consequently impairing fertility. Given that oocyte-specific DNA methylation establishment at imprinted genes occurs during this growth phase, we determined whether compromised endocrine signaling and gap junctional communication would disrupt *de novo* methylation acquisition. Individual oocytes from *Esr2-* and *Gja4*-deficient mice were assessed for DNA methylation establishment at *Snrpn, Peg3*, and *Peg1*. We observed no aberrant or delayed acquisition of DNA methylation at *Snrpn, Peg3*, or *Peg1* in oocytes from *Esr2-*deficient females, and no perturbation in *Snrpn* or* Peg3* *de novo* methylation in oocytes from *Gja4*-null females. However, *Gja4* deficiency resulted in a loss or delay in methylation acquisition at *Peg1*. One possible explanation for this difference between the three loci analyzed is the late establishment of DNA methylation at the *Peg1* gene. These results indicate that compromised fertility though impaired intercellular communication can lead to imprinting acquisition errors. Further studies are required to determine whether subfertility/infertility originating from impaired signaling and intercellular communication during oogenesis has an effect post-fertilization on imprint maintenance in the preimplantation embryo.

### GENE-SPECIFIC METHYLATION ACQUISITION ACCORDING TO OOCYTE SIZE

Our study is the first to investigate imprint methylation acquisition of *Snrpn,* *Peg3*, and *Peg1* in individual oocytes. We observed that each gene has its own size-dependent acquisition kinetics. *Snrpn* had the shortest acquisition interval with *de* *novo* methylation beginning at ~50 μm and near completion at >60 μm. *Peg3* had the earliest and longest acquisition interval. DNA methylation acquisition was initiated at ~45 μm and was nearly complete at >65 μm. *Peg1* had the latest acquisition of *de novo* methylation, beginning at ~55 μm and near completion by >70 μm. Previous studies reported similar findings using pooled oocytes where methylation level increased with days postpartum, follicular stage or with oocyte diameter/size, and initiation of acquisition was gene-specific (Lucifero et al., 2004; Hiura et al., 2006; Sato et al., 2007; Song et al., 2009). Oocyte-specific *de novo* methylation was also found to occur differentially with the maternal allele acquiring methylation prior to the paternal allele for *Snrpn*, *Zac1*, and *Peg1* (Lucifero et al., 2004; Hiura et al., 2006). Our data are consistent with this observation. Firstly, in oocytes for which two alleles were successfully amplified, one allele possessed higher and the other allele lower methylation levels, indicative of maternal and paternal contribution, respectively. For example, *Snrpn* WT563 oocyte had 81 and 50% methylation (**Figure 1**). Secondly, for oocytes within each diameter range (see *Peg3* control oocytes between 60 and 65 μm; **Figure 3**), a subset of oocytes had high methylation percentages (68, 71, 87, and 96%, indicative of the maternal allele) while others had low methylation percentages (18, 28, 48, 52%, indicative of the paternal allele). Finally, scatter plots show two distinct cohorts within the same range of diameter measurements. For example, *Peg1* control oocytes between 55 and 65 μm grouped into 0–40% methylation and 75–100% methylation (**Figure 6**).

### COMPROMISED FERTILITY LEADS TO LOSS OR DELAYED *Peg1* METHYLATION ACQUISITION

While *Gja4*-deficient oocytes ceased development and did not achieve mature size, our analyses indicated that they were not compromised in their ability to catalyze DNA methylation as *de novo* DNA methylation was initiated for the *Snrpn* and *Peg3* imprinted genes. The failure to initiate *Peg1* methylation acquisition may simply be due to the fact that oocytes lacking CX37 never reach the size necessary for *de novo* methylation to commence at late-acquiring loci. However, control oocytes of comparable size (55–60 μm) displayed initiation of *de novo* *Peg1* methylation. This suggests that *Peg1* methylation acquisition was lost or delayed in mutant oocytes. Alternatively, CX37-null oocytes may have reduced stores of methyl donors or other metabolites required for DNA methylation that would normally be transported from granulosa cells to the oocyte via gap junctions. If this is the case, then there must have been sufficient availability of methyl donors in mutant oocytes for *Snrpn* and *Peg3 de novo* methylation, but oocytes lacking junctional coupling with the granulosa cells may have exhausted their methyl donors during oocyte growth, preventing *de novo* methylation at late-acquiring genes like *Peg1*. To investigate the requirement for methyl donors during follicle development, Anckaert et al. (2010) cultured preantral follicles in medium with low methyl donors. While this led to impaired antrum development and polar body formation, it did not impede the acquisition of DNA methylation at the *Snrpn* ICR and the *Peg3* DMR. However, a reduced level of DNA methylation was found at the *Peg1* DMR. This provides support for the argument that gap junctional communication provides important metabolites for DNA methylation acquisition. To better understand the mechanism leading to loss or delayed methylation acquisition, further studies are required to assess the level of methyl donors, amount of *S*-adenosylmethionine, and ability to carry out global and gene-specific methylation in 55–60 μm CX37-null or CX37-depleted oocytes. Furthermore, methylation studies should be carried out using F_1_ females. For *Peg1* CX37 oocytes between 45 and 60 μm, oocytes possessed 0–53% methylation. DNA methylation acquisition was likely initiated on the maternal *Peg1* allele in some oocytes, while other oocytes lacked methylation on both parental alleles. Thus, loss or delayed *Peg1* methylation acquisition may preferentially lead to a failure of the paternal allele to become methylated. Further studies are required to investigate this potential grandpaternal effect.

*Peg1* may also be more susceptible to perturbation by assisted reproductive technologies. Loss of *Peg1/PEG1* methylation was observed in mouse oocytes following *in vitro* maturation (Kerjean et al., 2003), and human oocytes following ovarian stimulation (Sato et al., 2007). Further studies are required to determine whether the susceptibility of *Peg1* to perturbation** relates** to its late acquisition of methylation or whether a different epigenetic regulatory mechanism(s) operates at this gene. Superovulation also caused** imprinting errors in the mouse preimplantation embryo (Market-Velker et al., 2010), although imprinted methylation acquisition was not perturbed in mouse oocytes by exogenous hormone treatment (Anckaert et al., 2009; Denomme et al., 2011). We hypothesized that superovulation disrupts maternal-effect gene products required for imprint maintenance during embryo development. Thus, impaired fertility may not only disrupt *Peg1* methylation acquisition but may also lead to inadequate stores of maternal products, including those from granulosa cells, that may disrupt imprint maintenance at *Peg1* as well as at* Snrpn* and *Peg3* during preimplantation development. Extending studies to preimplantation embryos generated from fertilized ERβ-deficient and CX37-depleted oocytes will be required to determine their effects on imprint maintenance. In addition, further studies are required to determine whether assisted reproductive technologies, such as *in vitro* oocyte maturation and superovulation, lead to aberrant endocrine and paracrine signaling as well as granulosa cell–oocyte gap junctional communication.

It is important to understand granulosa cell–oocyte communication as technological advances move forward. Procedures such as slow-freezing cryopreservation and ultra-fast vitrification of oocyte-enclosed follicles, which employ cryoprotectants and very low temperatures, may permanently or temporally disrupt actin- or tubulin-rich projections that extend from granulosa cells through the zona pellucida to the oocyte (Kidder and Mhawi, 2002). Slow-freezing of mouse, rhesus monkey, and human preantral follicles disrupted projections and uncoupled the oocyte and granulosa cells (Barrett and Albertini, 2010). Temporal disruption of oocyte–granulosa cell contacts was also observed following vitrification (Trapphoff et al., 2010). Thus, transfer of molecules between the two compartments may be temporarily disturbed. While low levels of imprinting errors were detected in a subset of oocyte pools following vitrification (Trapphoff et al., 2010), further studies are required to determine whether disruption of oocyte–granulosa coupling leads to errors in imprint acquisition and/or maintenance.

Continued studies in animal models and in humans are required to understand the molecular mechanisms regulating genomic imprinting acquisition and maintenance as well as how impaired fertility and assisted reproductive technologies induce epigenetic changes and disease.

## Conflict of Interest Statement

The authors declare that the research was conducted in the absence of any commercial or financial relationships that could be construed as a potential conflict of interest.
